# Brain–Computer Interfaces in Parkinson’s Disease Rehabilitation

**DOI:** 10.3390/biomimetics10080488

**Published:** 2025-07-23

**Authors:** Emmanuel Ortega-Robles, Ruben I. Carino-Escobar, Jessica Cantillo-Negrete, Oscar Arias-Carrión

**Affiliations:** 1 División de Investigación en Neurociencias Clínica, Instituto Nacional de Rehabilitación Luis Guillermo Ibarra Ibarra, Mexico City 14389, Mexico; eortegar@inr.gob.mx (E.O.-R.); ricarino@inr.gob.mx (R.I.C.-E.); 2 Subdirección de Investigación Tecnológica, Instituto Nacional de Rehabilitación Luis Guillermo Ibarra Ibarra, Mexico City 14389, Mexico; jcantillo@inr.gob.mx; 3 Tecnologico de Monterrey, Escuela de Medicina y Ciencias de la Salud, Mexico City 14380, Mexico

**Keywords:** Parkinson disease, electroencephalography, brain–computer interfaces, neurorehabilitation, wearable electronic devices

## Abstract

Parkinson’s disease (PD) is a progressive neurological disorder with motor and non-motor symptoms that are inadequately addressed by current pharmacological and surgical therapies. Brain–computer interfaces (BCIs), particularly those based on electroencephalography (eBCIs), provide a promising, non-invasive approach to personalized neurorehabilitation. This narrative review explores the clinical potential of BCIs in PD, discussing signal acquisition, processing, and control paradigms. eBCIs are well-suited for PD due to their portability, safety, and real-time feedback capabilities. Emerging neurophysiological biomarkers—such as beta-band synchrony, phase–amplitude coupling, and altered alpha-band activity—may support adaptive therapies, including adaptive deep brain stimulation (aDBS), as well as motor and cognitive interventions. BCIs may also aid in diagnosis and personalized treatment by detecting these cortical and subcortical patterns associated with motor and cognitive dysfunction in PD. A structured search identified 11 studies involving 64 patients with PD who used BCIs for aDBS, neurofeedback, and cognitive rehabilitation, showing improvements in motor function, cognition, and engagement. Clinical translation requires attention to electrode design and user-centered interfaces. Ethical issues, including data privacy and equitable access, remain critical challenges. As wearable technologies and artificial intelligence evolve, BCIs could shift PD care from intermittent interventions to continuous, brain-responsive therapy, potentially improving patients’ quality of life and autonomy. This review highlights BCIs as a transformative tool in PD management, although more robust clinical evidence is needed.

## 1. Introduction

Brain–computer interfaces (BCIs), situated at the intersection of neuroengineering, systems neuroscience, and clinical medicine, are emerging as versatile platforms capable of restoring function, enhancing communication, and enabling adaptive neurorehabilitation. These systems typically extract brain signals—most commonly via non-invasive electroencephalography—and convert them into actionable outputs, thereby bypassing disrupted pathways of the peripheral nervous system. Enabled by advances in real-time signal processing, wearable biosensing, machine learning, and closed-loop feedback, BCIs are transitioning from experimental settings to therapeutic use, with applications spanning neurological and psychiatric disorders, including motor dysfunction, paralysis, and cognitive impairments; these cognitive domains include working memory deficits, attention disorders, executive dysfunction, slowed processing speed, age-related cognitive decline, and mood-related conditions such as depression, anxiety, and stress, which are particularly prevalent among older adults [1,2,3,4,5,6,7,8].

One condition for which these technological advances hold significant therapeutic promise is Parkinson’s disease (PD). It is a progressive neurodegenerative disorder defined by bradykinesia, rigidity, tremor, and a broad constellation of non-motor symptoms, for which current treatment strategies remain imperfect. Dopaminergic agents such as levodopa offer symptomatic relief, but motor fluctuations and dyskinesias frequently complicate long-term use. Deep brain stimulation (DBS), the most widely implemented neurosurgical intervention for PD, has expanded the therapeutic options, yet it is invasive, costly, and often restricted to a limited subset of patients. Non-pharmacological approaches—including physical and occupational therapies—can enhance functional capacity but demand sustained engagement and access, which are not always feasible [9,10,11,12,13].

Amid these challenges, BCIs represent a promising approach for decoding pathological brain activity and delivering personalized neuromodulatory interventions. These systems may enable monitoring of disease dynamics and activation of neuroplasticity mechanisms essential for functional recovery. In support of this potential, several electrophysiological biomarkers have been identified, providing real-time indicators of motor and cognitive dysfunction in PD. These include beta-band activity, phase–amplitude coupling, and alpha-band dynamics, features that can be detected through electroencephalography (EEG) or local field potentials [14,15,16]. As such, they represent key targets for BCI systems designed to tailor therapy, enable adaptive stimulation, and track disease progression in real time.

In this narrative review, we examine the potential of BCIs for clinical application in PD. We begin by outlining the fundamental components of BCIs, including current methods for signal acquisition, processing, and feature extraction. We then explore the neurophysiological characteristics of PD that are relevant for decoding brain states. Next, we review the clinical areas for which BCIs may offer benefits, with a focus on current literature on motor and non-motor symptom rehabilitation, adaptive neuromodulation, real-time monitoring, and domotic control. Finally, we discuss ongoing research and future directions, highlighting key challenges to broader implementation, such as algorithmic transparency, usability, and social acceptance.

Although initial studies regarding the use of BCIs in PD rehabilitation have shown promising results, significant barriers to clinical translation persist. First, while the neurophysiological substrates of alterations in PD are increasingly delineated, their reliable integration into responsive, real-world BCI systems remains a work in progress. Second, despite the efficacy of invasive neuromodulation, surgical risks and infrastructure demands limit scalability, underscoring the need for accessible, non-invasive alternatives. Third, the proliferation of consumer-grade wearable EEG devices has outpaced the development of regulatory and ethical standards, raising fundamental questions about the privacy of neural data, user autonomy, and health equity [17,18,19,20]. Finally, the complex interplay of motor, cognitive, and affective dysfunction in PD is rarely considered in existing BCI literature, which often prioritizes technical performance over clinical nuance.

Far from peripheral, the integration of BCIs into PD care represents a significant shift in how neurodegenerative disorders are conceptualized and managed. At the intersection of neuroscience, artificial intelligence, and bioelectronic medicine, these technologies have the potential to enhance therapeutic precision, increase patient engagement, and transform the field of neurorehabilitation.

## 2. Search Strategy

We conducted a literature search of articles published in peer-reviewed journals up to May 2025 using the PubMed, Web of Science, Scopus, IEEE Xplore, and Google Scholar databases. IEEE Xplore was included to ensure coverage of engineering and technical literature relevant to the development of BCI hardware, signal processing algorithms, and system integration, topics often underrepresented in biomedical databases. PubMed, Scopus, and Web of Science were selected to capture clinical, neuroscientific, and interdisciplinary studies relevant to Parkinson’s disease and BCI applications. These databases collectively offer a comprehensive overview of both the technical and clinical aspects of the field. The search included keywords such as “brain–computer interface”, “brain–machine interface”, and related terms, combined with “rehabilitation”, “therapy”, “treatment”, and “Parkinson’s disease”. No language restrictions were applied.

We included original clinical research studies—specifically clinical trials, case reports, and pilot studies—that investigated the use of a BCI or neural implant for the rehabilitation of motor or non-motor symptoms in patients with Parkinson’s disease. We excluded review articles, clinical protocols, and studies with poorly defined methodologies, lacking clinical outcome data, or insufficient information to support conclusions.

The initial database search yielded 489 articles. After removing duplicates, two researchers (O.A.-C. and E.O.-R.) independently screened the titles and abstracts of 373 records. Of these, 362 were excluded as irrelevant. An additional three relevant articles were identified through reference list screening. After full-text assessment for eligibility, 11 studies met the inclusion criteria and were included in this review. Additional details on the article selection process are provided in the Supplementary Material.

Furthermore, to obtain a perspective on the ongoing research on BCIs in PD rehabilitation, we searched ClinicalTrials.gov using “Parkinson’s Disease” as the condition/disease term, along with the following intervention/treatment terms: “brain–computer interface”, “neurofeedback”, “brain–machine interface”, “closed-loop deep brain stimulation”, and “adaptive DBS”.

## 3. Principles of Brain–Computer Interfaces

The development of BCIs represents a promising frontier in managing PD, enabling real-time decoding of cortical activity to drive therapeutic responses. These systems acquire information from the central nervous system, extract meaningful features, and translate them into digital commands to control external devices, such as rehabilitation and assistive technologies [17].

### 3.1. Signal Acquisition

BCIs can be broadly classified into invasive and non-invasive modalities based on the method of signal acquisition (Table 1). Invasive BCIs involve the surgical implantation of electrodes into or onto the brain, allowing for the direct recording of neural activity with high spatial and temporal resolution. Techniques such as electrocorticography (ECoG) and local field potentials (LFPs), which are commonly used, offer stable, high-fidelity signals that allow for the precise control of external devices [21,22,23]. Additionally, implanted electrodes can serve a dual purpose by enabling neural stimulation, as seen in applications like deep brain stimulation for movement disorders or epilepsy [24]. However, their use involves risks related to the surgical procedure itself, as well as potential long-term biocompatibility issues. Additionally, the long-term durability of invasive electrodes is limited, as they can trigger an inflammatory response and reactive gliosis, ultimately leading to electrode encapsulation and reduced signal acquisition accuracy [7]. In contrast, non-invasive BCIs acquire brain signals using external sensors that do not physically penetrate the skull, making them safer and more accessible; however, they typically suffer from lower spatial or temporal resolution and greater susceptibility to noise. These include methods such as EEG, functional near-infrared spectroscopy (fNIRS), functional magnetic resonance imaging (fMRI), magnetoencephalography (MEG), and transcranial Doppler ultrasonography (TCD), each offering unique advantages in terms of portability, coverage, and compatibility with various cognitive and motor tasks [4,25].

The comparative assessment presented in Table 1 uses a symbolic scale to qualitatively (“+++” to “− −”) rank various BCI signal acquisition methods across parameters such as long-term recording suitability, portability, cost, and safety. These rankings are not derived from a standardized metric but instead reflect the authors’ synthesis of current literature and expert opinion [4,7,23,25,26,27]. Each symbol reflects a consensus observed in the literature—for example, EEG is typically described as highly portable (+++), low in cost (−), and very safe (+++), while fMRI offers excellent spatial resolution but is limited by low portability (− −) and high cost (+++). These ratings are intended to support comparative understanding and are not meant to be exhaustive or prescriptive, as actual performance characteristics may vary depending on device specifications, clinical context, and user needs.

### 3.2. Non-Invasive Electrode Technologies for EEG-Based BCIs

EEG remains the most widely used signal acquisition modality in non-invasive BCI systems for rehabilitation due to its millisecond-level temporal resolution, low cost, portability, and ease of use, making it well suited for real-time applications. In most BCI systems, scalp electrodes are placed according to the standard 10–20 international system, which provides adequate spatial coverage for many applications, although high-density arrays are increasingly employed in research settings [4].

Non-invasive EEG electrodes are typically categorized into three types: wet, dry, and non-contact electrodes [23].

Wet electrodes are the conventional type and rely on conductive gels to establish low-impedance contact with the scalp. They are made from materials such as silver/silver-chloride (Ag/AgCl), gold, or stainless steel. These electrodes may be disposable or reusable and are often used in combination with adhesive matter or fixed to specialized caps to improve signal stability. While they provide high-quality signals, they require time-consuming preparation and can cause discomfort due to the application of gel.

Dry electrodes were developed to address the main limitation of wet electrodes by eliminating the need for gels or skin preparation. Designs include spike-array (comb-shaped) electrodes with microscale pillars made from materials like silicon, carbon nanotubes, or flexible conductive materials, e.g., silver-coated polymers or titanium/gold coatings fabricated via 3D printing, which offer improved comfort and ease of use. These electrodes use an active design that incorporates signal preamplification components within the electrode housing. However, signal quality and stability can vary depending on the electrode design and application and are generally reported to be lower than those of wet electrodes [26].

Non-contact electrodes use capacitive coupling to detect EEG signals without direct contact with the skin. These electrodes significantly reduce setup time and improve user comfort but often suffer from low signal amplitude and are more susceptible to motion artifacts. To enhance performance, amplifier-integrated designs that incorporate on-board signal processing (e.g., amplification, filtering, and digitization) have been introduced. Some systems are now fully wireless, facilitating portable and wearable EEG monitoring [23,26,27].

### 3.3. Signal Processing

In EEG-based BCI systems (eBCI), online signal processing plays a critical role in ensuring accurate and reliable interpretation of neural activity. The processing pipeline typically begins with preprocessing, which aims to enhance signal quality by removing noise and artifacts that can interfere with the assessment of brain signals. Artifact removal is critical given the susceptibility of EEG signals to various sources of interference. These artifacts may be physiological (e.g., eye blinks, muscle activity, cardiac signals) or non-physiological (e.g., poor electrode contact, environmental noise). Several techniques are used to address these issues, including digital filtering to isolate specific frequency bands, independent component analysis (ICA) to separate and remove sources of noise, wavelet transform (WT) for time-frequency decomposition, canonical correlation analysis (CCA) to reduce electromyographic contamination, and empirical mode decomposition (EMD) for handling the non-linear and non-stationary nature of EEG data [28]. In recent years, deep learning (DL) approaches, such as convolutional neural networks (CNNs), generative adversarial networks (GANs), and diffusion models, have shown promise in automating artifact removal with high precision by directly learning complex noise patterns from the data [25,28,29]. Most of these techniques have been implemented for online artifact removal, with the notable exception of EMD, which remains computationally demanding and is typically applied offline [30].

Following preprocessing, feature extraction is performed to identify and isolate patterns in the EEG signals that are informative of user mental states—such as motor intentions or cognitive processes—or to detect pathological neural activity relevant for BCI control and therapeutic feedback. This reduces data dimensionality while preserving critical information. Commonly extracted features include time-domain features (e.g., statistical measures, entropy, signal energy), frequency-domain features (e.g., power spectral density, band-specific metrics), and time–frequency-domain features derived from methods such as short-time Fourier transform, WT, and Hilbert–Huang transform. In addition, spatial-domain features, such as those obtained using common spatial pattern (CSP) analysis, are widely used to enhance signal separability across different brain states. Deep learning models also contribute to feature extraction by learning task-relevant representations from raw or minimally processed data. These models can be designed to function in a discriminative, representative, generative, or hybrid manner, depending on the specific requirements of the BCI application [25,28,29].

After feature extraction, a classification stage is required to translate the extracted EEG features into control commands or diagnostic outputs. EEG-based BCI systems utilize a range of machine learning algorithms, including linear discriminant analysis, quadratic discriminant analysis, support vector machines, k-nearest neighbors, naive Bayes, decision trees, random forests, and Gaussian mixture models. Riemannian geometry-based classifiers are also widely used, particularly for motor imagery tasks. More recently, deep learning approaches such as convolutional neural networks, recurrent neural networks, long short-term memory networks, deep belief networks, and autoencoders have shown promise for automatically learning complex features from raw EEG signals. The choice of classifier depends on factors such as signal characteristics, computational resources, and the specific BCI application [31,32].

The performance of machine learning algorithms is typically evaluated using metrics derived from the confusion matrix, including accuracy, precision, recall (also known as sensitivity), specificity, and the F1 score. For imbalanced datasets or multi-class problems, additional measures such as Cohen’s kappa, the Matthews correlation coefficient, and area under the ROC curve are often used. These metrics help determine how well the algorithm distinguishes between relevant brain states or user intentions, which is critical for reliable BCI operation [33,34].

Although the signal processing techniques described for EEG are not directly transferable to other acquisition modalities, such as fMRI or fNIRS, the core objectives—artifact removal, dimensionality reduction, feature extraction, and classification—are consistent across modalities. Each signal type presents distinct characteristics that require tailored processing strategies. Nevertheless, the selection and optimization of signal processing methods remain critical to the performance of BCI systems and continue to be a major focus of ongoing research.

### 3.4. Feedback Mechanisms in BCI-Based Rehabilitation

In rehabilitation, BCIs commonly employ feedback mechanisms such as functional electrical stimulation (FES), robotic exoskeletons, and virtual reality (VR) to support motor recovery. FES activates specific muscles in response to detected motor intentions, while exoskeletons assist in performing goal-directed movements. VR environments offer immersive, interactive scenarios that enhance engagement and neuroplasticity. Other feedback modalities include auditory cues, visual displays (e.g., moving cursors or avatars), tactile stimulation (e.g., vibrations), augmented reality, and neurofeedback based on EEG rhythms. Together, these techniques form closed-loop systems that facilitate brain reorganization and functional improvement [4].

### 3.5. Common eBCI Paradigms

BCI paradigms, which refer to the strategies used to convert specific patterns of brain activity into control signals, are commonly categorized based on whether they depend on evoked or spontaneous neural activity (Table 2) [25,29,35,36]. *Evoked paradigms*, such as steady-state visual evoked potentials (SSVEPs) and P300 event-related potentials, depend on time-locked responses to external stimuli. These paradigms typically offer high signal-to-noise ratios, fast calibration, and relatively high information transfer rates [37,38,39]. However, they require preserved visual or auditory function and sustained attention, which may limit their use in individuals with sensory deficits or cognitive fatigue [1,17,40]. Evoked paradigms are more commonly applied in assistive communication and control interfaces, while their use in rehabilitation is less frequent.

In contrast, spontaneous paradigms are based on endogenous brain activity, including motor imagery (MI) and slow cortical potentials (SCPs). These approaches capitalize on internally generated neural signals and often align with the intrinsic physiology of motor planning and volitional control [18,25,40]. MI-based BCIs, for example, allow users to control external devices by imagining specific movements, without executing actual motor output. This strategy leverages preserved sensorimotor rhythms, particularly in the α (8–13 Hz) and β (13–30 Hz) frequency bands, and has demonstrated promise in applications such as upper-limb rehabilitation, gait initiation, and neurorehabilitation in patients with stroke, spinal cord injury, or neurodegenerative diseases [40,41,42].

SCP-based BCIs rely on detecting slow voltage shifts in cortical activity and are typically used for binary control applications. These systems require more extensive user training but can be effective for individuals with severe motor impairments, including those with locked-in syndrome, freezing of gait, or anarthria [29,35,43].

Finally, hybrid paradigms—which combine EEG with additional modalities such as electromyography (EMG), eye-tracking (EOG), or inertial sensors—are increasingly used to enhance signal reliability and classification performance. These systems offer multimodal redundancy, which is particularly valuable in populations with fluctuating motor or cognitive function and can help mitigate the impact of artifacts or transient signal loss [25,29,36].

### 3.6. Toward Clinical Integration

Despite rapid progress in eBCI technology, clinical adoption remains limited. Future systems must prioritize portability, user-centered design, and seamless integration with standard-of-care treatments to bridge this gap. Wireless EEG systems and wearable biosensors are increasingly capable of performing continuous monitoring in home settings, facilitating real-world data collection, supporting longitudinal symptom tracking, and enabling remote therapeutic adjustments.

Integrating digital health platforms, such as smartphone-based apps or telemedicine dashboards, could expand accessibility and patient engagement. Clinical deployment must also consider training protocols, caregiver involvement, and mechanisms for monitoring system safety over time.

## 4. Neurophysiological Correlates of Parkinson’s Disease Relevant to BCI

Parkinson’s disease is increasingly recognized as not merely a motor disorder but as a complex, multisystem neurodegenerative condition involving widespread dysfunction across cortical, subcortical, and brainstem circuits. Beyond the dopaminergic system, alterations in glutamatergic, GABAergic, and cholinergic pathways contribute to a wide range of motor and non-motor symptoms, including cognitive impairment, mood disturbances, and autonomic dysfunction [9]. The diverse manifestations of the disease are reflected in dynamic changes in brain activity that can be detected through neurophysiological and neuroimaging techniques—tools essential for developing BCI systems tailored for PD.

EEG is a non-invasive method particularly well-suited to monitoring the large-scale oscillatory dynamics disrupted in PD. EEG signals primarily reflect synchronized postsynaptic potentials in cortical pyramidal neurons, making them a valuable window into network-level brain activity [3,17]. In PD, pathological synchronization within the cortico–basal ganglia–thalamocortical loops is a key feature, especially in the beta frequency band (13–30 Hz). Excessive beta-band activity, as recorded from subthalamic nucleus (STN) LFPs, is strongly associated with motor symptoms such as rigidity and bradykinesia [24,44,45]. Moreover, beta oscillations in the STN exhibit phase-amplitude coupling (PAC) with high-frequency activity (200–400 Hz), and the strength of this coupling correlates with clinical severity scores, such as the UPDRS akinesia–rigidity subscale [46]. These pathological oscillations have become diagnostic markers and targets for adaptive neuromodulation, including closed-loop deep brain stimulation protocols [24]. Importantly, pathological beta-band activity also demonstrates coupling between subcortical and cortical structures, allowing its detection via scalp EEG. This cortical–subcortical coherence supports the feasibility of real-time BCI systems that can operate non-invasively and dynamically adapt to pathological neural states [47].

From a clinical perspective, these neurophysiological markers are increasingly used to guide therapeutic strategies. For instance, while DBS remains highly effective in reducing motor symptoms in selected patients, its efficacy is limited by the static nature of open-loop stimulation and the need for extensive calibration. LFP-based BCI systems, by contrast, offer the potential for dynamic and adaptive stimulation—modulating neural circuits based on real-time cortical activity. This transition toward closed-loop paradigms will likely enhance therapeutic precision and reduce off-target effects [24,48].

Despite its utility, beta power alone has shown inconsistent responses to medication in scalp EEG studies, limiting its reliability as a standalone biomarker [46]. In contrast, beta bursts—transient increases in beta activity—have been shown to decrease in frequency during the OFF-medication state and are linked to symptom severity [49]. Another well-established biomarker is the excessive PAC between beta phase and broadband gamma (50–200 Hz) amplitude, which tends to decrease with medication and shows spatial specificity to sensorimotor regions [50]. Similarly, the asymmetry in beta waveform shape has been correlated with PAC strength and is also reduced by therapy [51]. More recently, nonlinear dynamics in the alpha band (8–12 Hz) have gained attention. A measure of nonlinear autocorrelated memory in the alpha band has been shown to increase with dopaminergic medication and is significantly lower in unmedicated PD patients compared to in healthy controls. This alpha-band nonlinearity is spatially constrained to sensorimotor areas and provides a robust, noninvasive EEG biomarker that complements beta-derived features, potentially offering a valuable tool for detecting early-stage PD [46].

Another domain with growing relevance for BCI applications is motor imagery. MI tasks engage cortical circuits through the mental rehearsal of movement, activating sensorimotor networks even in the absence of overt motion. This approach holds promise for enhancing neuroplasticity and functional recovery during rehabilitation, even in individuals with severe motor impairment [40,52], making MI-based paradigms attractive for rehabilitation in PD, especially for patients with severe physical limitations due to bradykinesia, rigidity, or fatigue [53].

A key structure in this context is the supplementary motor area (SMA), which plays a crucial role in movement initiation. In PD, dopaminergic depletion in the dorsal striatum leads to excessive thalamic inhibition and consequently, to reduced excitatory drive to the SMA. Dysfunction of the SMA is implicated in akinesia, and although functional imaging studies have shown variable results, most report reduced SMA activation during voluntary movement. As such, SMA activity has become a target for EEG-based neurofeedback therapies and BCIs aimed at restoring movement initiation in PD [54].

In summary, biomarkers ranging from EEG dynamics to functional SMA activity offer a rich set of physiological signals that can inform the design of personalized, real-time BCIs for individuals with PD, not only for symptom monitoring and adaptive neuromodulation but also for enhancing neurorehabilitation through strategies such as motor imagery and neurofeedback.

## 5. Current and Emerging Applications of BCIs in Parkinson’s Disease

**Figure 1:**
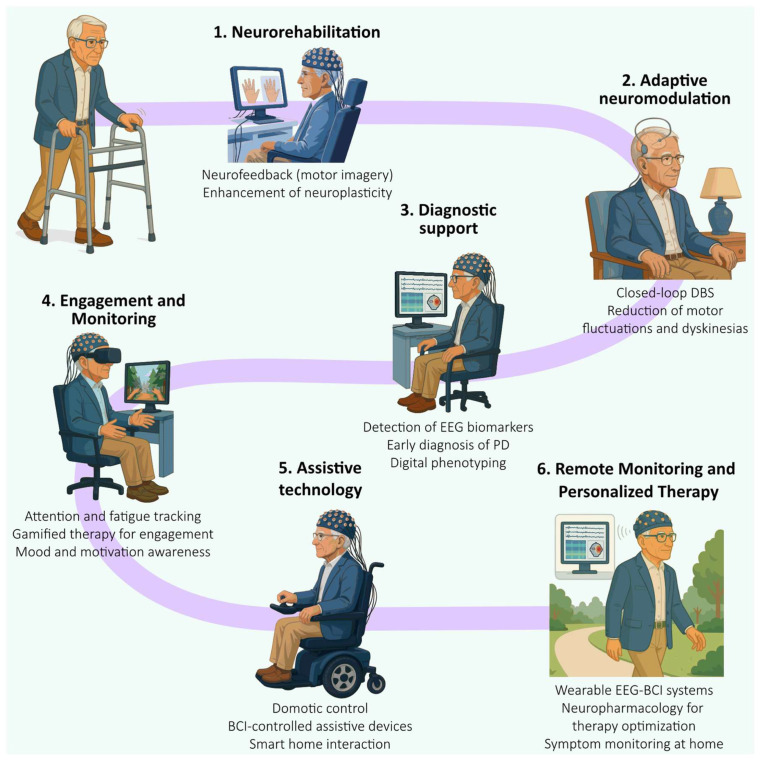
Current and emerging applications of BCIs in Parkinson’s disease. Applications span six key domains: (**1**) neurorehabilitation, where neurofeedback using motor imagery may enhance neuroplasticity and functional recovery of motor and non-motor symptoms; (**2**) adaptive neuromodulation, with closed-loop deep brain stimulation (DBS) dynamically adjusting therapy based on neural activity; (**3**) diagnostic support, including the detection of EEG biomarkers for early diagnosis, and digital phenotyping; (**4**) engagement and monitoring, leveraging cognitive state tracking and gamified therapy to promote adherence and well-being; (**5**) assistive technology, enabling BCI-controlled wheelchairs and smart home interactions; and (**6**) remote monitoring and personalized therapy, using wearable eBCIs to optimize treatment and track symptoms at home. These systems reflect a shift toward non-invasive, patient-tailored neurotechnology designed to enhance quality of life and clinical outcomes in PD.

Initially developed for communication in locked-in syndrome, BCIs have evolved into multi-purpose tools capable of addressing motor, cognitive, and psychosocial deficits across a range of neurological conditions. Integrating eBCIs into therapeutic and diagnostic pathways in PD reflects a critical shift toward patient-specific, non-invasive technologies. These systems leverage cortical oscillatory biomarkers to enable personalized neurorehabilitation, adaptive neuromodulation, real-time symptom tracking, and autonomous domotic interaction (Figure 1).

### 5.1. Neurorehabilitation and Therapeutic Modulation

Rehabilitation strategies in PD are undergoing a significant evolution, transitioning from fixed, therapist-driven regimens to closed-loop, user-adaptive systems [55]. BCIs aim to directly detect transient brain dynamics and utilize them as part of the therapy, supporting neuroplastic reorganization and functionally meaningful recovery.

Our search of digital databases identified only 11 studies that used a BCI for the rehabilitation or management of motor or non-motor symptoms in PD (Table 3). A notable approach emerging in this field is adaptive deep brain stimulation (aDBS), a form of closed-loop stimulation that adjusts its parameters in response to ongoing neural activity. Several studies demonstrate the feasibility and benefits of this technique. For instance, Little et al. provided a proof-of-principle that BCI-controlled aDBS using STN-LFPs as feedback can improve motor symptoms more effectively and efficiently than conventional continuous DBS, achieving better clinical outcomes with reduced stimulation time and energy requirements [56]. Follow-up work by the same group extended these findings to bilateral aDBS, showing significant improvement in both limb and axial symptoms, even when patients were concurrently taking medication [57].

Similarly, Arlotti et al. confirmed the clinical efficacy of LFP-guided aDBS during daily activities, reporting it to be safe and well-tolerated and observing improvements in motor function and reduced dyskinesias, further validating its real-world applicability [58]. A more recent study introduced the AlphaDBSR system, an implantable closed-loop neural interface that supports artifact-free neural recordings and long-term data management. This system was successfully applied in PD patients as a clinically viable BCI for aDBS, demonstrating the potential for chronic therapeutic use [44].

Other research has focused on improving the technological infrastructure for BCI-based neuromodulation. Dold et al. presented Dareplane, an open-source, modular software platform that facilitates the implementation of BCI setups, including aDBS experiments. They demonstrated its feasibility in both laboratory-based evaluations and in a closed-loop session with a PD patient using externalized DBS leads. The platform also supported a non-invasive BCI speller, underscoring its flexibility across different neurotechnological applications [59].

Clinical application of fully implantable bidirectional neural interfaces has also progressed. Swann et al. used the Activa PC + S system to chronically record cortical and subthalamic LFPs in PD patients over one year while delivering therapeutic DBS. This study demonstrated the long-term stability and safety of such implants, as well as their utility in tracking movement-related neural dynamics, which supports their potential role in next-generation neuromodulation therapies [60].

Another advancement comes from Velisar et al., who implemented a dual-threshold neural closed-loop DBS (NclDBS) system using the Activa PC + S in PD patients with chronic stimulation. Their beta-band-driven algorithm significantly improved tremor and bradykinesia, while delivering nearly half the electrical energy required by conventional DBS, highlighting the efficiency and personalization possible with NclDBS [61].

In addition to stimulation-based interventions, functional neuroimaging-based BCIs have also shown promise. Subramanian et al. used real-time fMRI neurofeedback to train PD patients to upregulate activity in the SMA using motor imagery. This intervention led to improvements in motor speed and clinical motor ratings, demonstrating that patients can learn to volitionally modulate motor circuitry, with beneficial effects not observed in a control group lacking feedback [62]. Similarly, Buyukturkoglu et al. reported that fMRI-based BCI training targeting the SMA affected motor performance in PD, suggesting that altered activation patterns in this region are modifiable through volitional control and may be therapeutically relevant [54].

Lastly, preliminary clinical applications of BCI-based neurorehabilitation targeting both motor and non-motor impairments in PD have been explored. Lavermicocca et al. applied EEG-based neurofeedback using the NeuroSky MindWave headset in a cohort of PD patients with mild cognitive impairment. Over 24 sessions, patients demonstrated significant cognitive improvements and reported high levels of satisfaction, suggesting that neurofeedback may empower patients through enhanced self-regulation of brain activity [63]. Turconi et al. evaluated a motor imagery-based BCI protocol in three PD patients across 15 sessions. Improvements were noted in gait (particularly freezing episodes), EEG alpha and beta power, and performance on attention and executive function tasks, indicating that BCI tools may be integrated into rehabilitative strategies for both motor and cognitive symptoms [64].

**Table 3:** Studies of BCI-based rehabilitation in Parkinson’s disease.

Study	Intervention	Time	Sample Size	Main Findings
Turconi et al., 2014 [64]	EEG-BCI neurofeedback (motor imagery) for motor and cognitive rehabilitation.	15 sessions, 2–3 times per week	3 PD	Decrease in severity of gait freezing, improvement in mobility, increase in alpha and beta EEG bands power, and better performance on attention and executive tasks.
Lavermicocca et al., 2018 [63]	EEG-BCI neurofeedback (attentional control) for cognitive rehabilitation.	24 sessions in 3 months	10 PD	Cognitive performance increased compared to baseline in all cognitive domains (attention, set shifting, executive functions, verbal fluency, immediate and delayed auditory-verbal memory, and visual–spatial reasoning), with a positive impact on reaction time, processing speed, and overall efficiency.
Subramanian et al., 2011 [62]	fMRI-BCI neurofeedback (motor imagery) for hand motor rehabilitation.	2 BCI sessions and 2 to 6 months of neurofeedback practice at home	5 PD	Improvement in motor speed (finger tapping) and clinical ratings of motor symptoms (37% in UPDRS part III).
Buyukturkoglu et al., 2013 [54]	fMRI-BCI neurofeedback (motor imagery), plus a motor task for hand motor rehabilitation.	1 session	1 PD 3 HS	Hand motor responses slowed down.
Little et al., 2013 [56]	BCI-controlled adaptive DBS (unilateral).	640 s	8 PD	Improved motor scores (UPDRS) and reduction in stimulation time and energy requirements compared to those of conventional DBS.
Little et al., 2016 [57]	BCI-controlled adaptive DBS (bilateral).	15 min	4 PD	Motor scores showed improvement compared to those in the absence of stimulation.
Arlotti et al., 2018 [58]	BCI-controlled adaptive DBS (unilateral).	8 h	11 PD	Motor scores showed improvement compared to those in the absence of stimulation.
Swann et al., 2018 [60]	BCI-controlled adaptive DBS (multisite brain recordings, bilateral stimulation).	–	5 PD	Four of the five patients showed improved motor function 1 year postoperatively.
Velisar et al., 2019 [61]	BCI-controlled adaptive DBS (bilateral, Activa™ PC + S-NexusD3).	21 min	13 PD	Closed-loop DBS was feasible, well-tolerated, and improved tremor and bradykinesia, reducing energy requirements.
Arlotti et al., 2021 [44]	BCI-controlled adaptive DBS (AlphaDBS System).	24 h; then 2 weeks	3 PD	The implanted BCI was viable for adaptive DBS with artifact-free and long-term recordings.
Dold et al., 2025 [59]	BCI-controlled adaptive DBS (Dareplane) for research.	23 min	1 PD	The system was viable for adaptive DBS.

Abbreviations: PD, Parkinson’s disease; HS, healthy subjects; EEG, electroencephalography; fMRI, functional magnetic resonance imaging; BCI, brain–computer interface; UPDRS, Unified Parkinson’s Disease Rating Scale; DBS, deep brain stimulation.

The integration of BCIs and neural implants into PD rehabilitation is showing promising potential. Adaptive DBS systems have demonstrated superior clinical outcomes and energy efficiency, while neurofeedback and neuroimaging-guided BCIs offer non-invasive options for modulating motor circuits [44,56,57,58,59,60,61]. The development of robust, patient-tailored neural interfaces represents a significant step toward more effective and individualized care for PD.

### 5.2. Diagnostic Potential and Disease Monitoring

While BCIs are not currently used as standalone diagnostic tools for PD, the techniques developed within BCI research—such as non-invasive neural signal acquisition, advanced signal processing, and machine learning methods—are being leveraged to improve the detection and monitoring of PD-related neurophysiological changes. For example, excessive beta synchrony and increased gamma activity in the STN, recognized neurophysiological biomarkers of motor dysfunction [14,24], can be detected using BCI-related techniques. These signals may help track motor and cognitive aspects of disease progression and support therapeutic decision making.

eBCIs can help distinguish cognitive decline in PD from other dementias by revealing specific oscillatory patterns. PD patients with mild cognitive impairment (MCI) show increased theta and reduced beta power, along with lower alpha/theta and alpha2/alpha1 ratios, compared to those with Alzheimer’s disease-related MCI and healthy controls. These features, which correlate with cognitive performance, may aid in diagnostic classification and monitoring of cognitive decline in PD [15]. A recent meta-analysis identified 59 studies that applied EEG analysis combined with machine learning for the diagnosis of PD, the classification of cognitive impairment, and the prediction of gait abnormalities [16].

Neuropharmacology is another emerging use case. By analyzing spectral changes in response to dopaminergic therapy or DBS, clinicians can monitor treatment response, guide dosage adjustments, and identify adverse reactions in real time [65,66]. This is especially valuable in advanced stages, where medication efficacy becomes unpredictable, and wearable EEG may enable adaptive management in the home setting.

### 5.3. Domotic Control and Daily Function

Restoring autonomy in daily activities is a key therapeutic goal in PD. eBCIs enable control of wheelchairs, lighting, appliances, and communication systems through intuitive brain signals—reducing dependence on caregivers during OFF periods or freezing episodes [1,2,40]. These systems can interpret simple motor intentions or sustained attention levels to trigger predefined actions, allowing users to maintain interaction with their environment despite severe motor impairments.

In occupational settings, wearable EEG is being tested to monitor cognitive load, fatigue, and alertness in real time [6,29,36]. Similar approaches could mitigate fall risk or therapy dropout in PD. Adaptive DBS algorithms could also incorporate fatigue-related data to tailor stimulation protocols.

### 5.4. Education, Engagement, and Neurocognitive Stimulation

Cognitive symptoms—including executive dysfunction, slowed processing, and impaired working memory—affect a substantial proportion of individuals with PD [67]. EEG biomarkers such as frontal theta (linked to effortful processing) and posterior alpha (related to memory encoding) can inform personalized cognitive training [68]. These biomarkers allow clinicians to tailor interventions and monitor effectiveness in real time [1,69]

Outside traditional rehabilitation, eBCIs are driving innovation in neuro-entertainment and digital engagement. Patients can interact with games, virtual reality environments, and media using mental commands or emotion-driven inputs—providing stimulation that is both therapeutic and enjoyable. These tools may be especially valuable in the early and moderate stages of PD, when cognitive engagement can delay decline and reduce social isolation [4,6,36,40].

Neuromarketing techniques derived from EEG research are also being repurposed in healthcare to assess emotional responses to communication, therapy adherence, and patient education materials. By decoding attentional and affective responses, these tools may enhance clinician–patient communication and treatment personalization [6,29].

EEG-based BCIs are transforming PD management by supporting motor recovery, emotional well-being, and daily autonomy through applications in rehabilitation, monitoring, and domotic control. To transition from research to routine clinical use, these systems require thorough validation, user-centered design, ethical oversight, and collaboration across sectors. When effectively implemented, eBCIs have the potential to restore lost functions and improve the quality of life for people with PD.

## 6. Design Considerations for BCIs in Parkinson’s Disease

The development of effective eBCIs for PD necessitates a multidisciplinary approach that carefully considers the unique physiological and behavioral challenges presented by this condition. While advances in signal processing and machine learning have significantly expanded the potential of BCIs, the reliability, usability, and clinical scalability of these systems still depend on key design factors, including electrode technology, signal quality, adaptability to fluctuating symptoms, and user-centered control strategies.

### 6.1. Electrode Technology and Signal Acquisition

Electrode performance is a critical determinant of the practicality and long-term utility of eBCIs. In PD, where symptoms such as tremor, dyskinesia, and variable skin conditions can disrupt stable recordings, traditional EEG electrodes are often inadequate. Issues such as impedance variability, motion-induced artifacts, and discomfort due to hair interference or pressure sensitivity are magnified in this population, particularly for patients undergoing dopaminergic therapy, which can alter skin characteristics [70,71,72,73].

To address these limitations, several innovative electrode designs have emerged. Tattoo-like thin-film sensors and brush-type fingered electrodes adapt better to the scalp’s irregular surface, improving both signal quality and user comfort [74,75]. Semi-dry electrodes, which employ small electrolyte reservoirs that hydrate upon skin contact, offer a pragmatic balance between performance and usability [76]. These designs reduce application time, improve wearability, and are better suited for prolonged use in home-based monitoring and telerehabilitation—an increasingly central component of PD care [3,9].

### 6.2. Signal Processing and Feature Extraction

The complexity of brain signals in PD necessitates robust preprocessing pipelines to eliminate artifacts and enhance the signal-to-noise ratio, ensuring the reliability of downstream analyses [3,40]. High-density EEG systems, although more complex, enable spatial filtering techniques that can exclude noisy peripheral channels and apply source or subspace reconstruction methods to isolate meaningful neural activity [70].

Feature extraction algorithms must capture disease-relevant neural dynamics and user intent. Frequency and temporal markers—especially those linked to beta-band oscillations and motor planning—are central to this task. Deep learning methods, while data-intensive, may offer the advantage of automated feature learning and reduced dependence on domain-specific tuning, thereby supporting the development of generalizable, plug-and-play systems suitable for real-world deployment [77,78]. These approaches are particularly relevant in PD, for which daily symptom fluctuations necessitate flexible algorithms that adapt to evolving brain states.

### 6.3. Adaptive and Inclusive System Design

Effective design of BCI systems for PD must consider the user’s motor limitations, cognitive capacity, fatigue, and motivation, all of which vary across both individuals and over time. Systems requiring simultaneous control of multiple commands may overwhelm users with advanced symptoms or cognitive impairment. To mitigate this, shared-control paradigms have been proposed. These systems enable users to specify general goals, while machine learning agents manage execution details, thereby reducing cognitive load, improving ease of use, and supporting long-term adherence [1,79]. Moreover, detecting non-control states—periods in which the user is disengaged—is critical for safety and system reliability. In response to this need, various strategies have been proposed in the literature. Idle-state classifiers identify neural patterns associated with rest or disengagement, helping to prevent unintended commands [80,81,82]. Brain-controlled switches enable users to activate or deactivate the BCI deliberately, offering an additional layer of intentional control [83]. Attention-level monitoring assesses the user’s cognitive engagement to ensure that commands are issued only when attention is sufficiently focused [84]. Together, these approaches help distinguish intentional actions from passive states, thereby minimizing the risk of accidental system activation.

### 6.4. Paradigm Selection and Feedback Integration

The choice of control paradigm also significantly influences BCI effectiveness. Evoked paradigms, which rely on intact sensory and attentional systems, may be suboptimal for individuals with PD-related dementia or visual deficits. In contrast, spontaneous paradigms that harness endogenous motor planning activity may align more naturally with the cortico–subcortical dynamics altered in PD and offer better long-term usability [85].

Incorporating feedback—tactile, auditory, or even cortical stimulation—into closed-loop systems fosters co-adaptation between the user and the BCI. These bidirectional systems not only improve real-time control but also support neuroplasticity and motor relearning, aligning with rehabilitation goals in PD [52,86,87].

### 6.5. Commercial Wearable EEG Systems: Opportunities and Limitations

Advances in wireless technology and low-power electronics have led to the development of consumer-grade wearable EEG devices, enabling continuous, non-invasive brain monitoring outside clinical settings. These systems, often using dry or semi-dry electrodes, offer new opportunities to track cognitive, motor, and sleep-related changes in individuals with PD. While promising for long-term monitoring of symptoms such as motor fluctuations and OFF episodes, most commercial devices lack the sampling rates, spatial resolution, and electrode flexibility needed for clinical-grade analysis—particularly in detecting subtle cortical features relevant to PD [88,89,90].

To improve signal quality, newer systems integrate active amplification at the electrode level, reducing ambient noise in real-world conditions [17]. In addition, hybrid approaches that combine EEG with EMG, EOG, or accelerometry are being developed to enhance system reliability and account for variability across users. These multimodal systems are especially valuable for individuals with severe motor symptoms or difficulty using standard BCIs, and they may support more precise, context-aware interactions in rehabilitation and assistive applications [1,18,29].

## 7. Current Research and Future Perspectives on BCIs for Parkinson’s Disease Rehabilitation

Recent advances in BCI technology are transforming the landscape of rehabilitation and assistive technologies for PD, offering novel approaches to monitor, modulate, and enhance motor and non-motor function. Evidence supporting the use of BCIs for PD treatment is still scarce, with outcomes from these interventions reported in only 64 patients with PD in total, as shown in Table 3. These studies reflect the emerging but still limited application of BCIs in Parkinson’s disease rehabilitation. Only a few studies have explored non-invasive BCI approaches using EEG or fMRI, mostly in small pilot trials focused on motor imagery and neurofeedback. Notably, no studies were identified that used other non-invasive modalities, such as fNIRS, MEG, or transcranial Doppler ultrasonography. In contrast, the majority of published research has centered on aDBS, indicating that current clinical interest is largely directed toward invasive, closed-loop neuromodulation systems.

**Figure 2:**
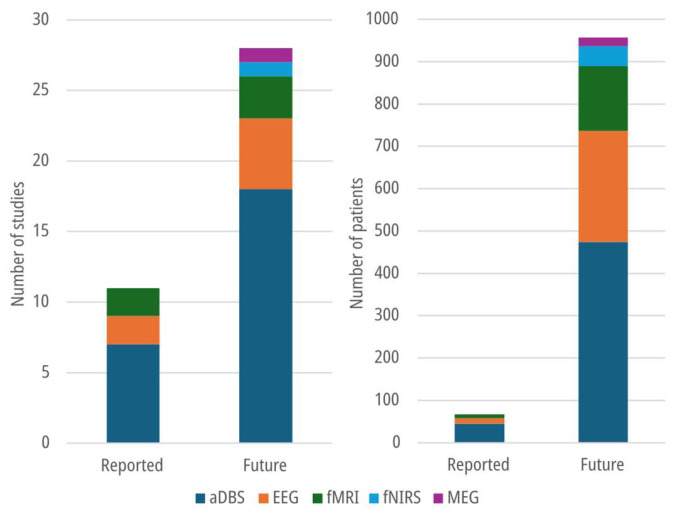
Current (reported) and projected (future) clinical research activity in BCI applications for Parkinson’s disease rehabilitation. The chart illustrates the number of published studies and estimated participant counts in comparison to the planned clinical trials registered in ClinicalTrials.gov. Data are grouped by BCI modality, including aDBS and neurofeedback approaches (EEG, fMRI, fNIRS, and MEG). This visualization illustrates the increasing clinical interest and anticipated expansion of the evidence base in the years to come.

A review of ClinicalTrials.gov reveals growing interest in this area (Table A1), with 29 ongoing trials investigating BCI applications in PD rehabilitation. Of these, 18 focus on aDBS, while the remaining studies explore non-invasive BCIs for neurofeedback and symptom management. These include systems based on EEG (five studies), fMRI (three studies), ECoG (one study), fNIRS (one study), and MEG (one study). Notably, the number of participants enrolled in upcoming trials suggests a significant expansion of evidence in the near future. For example, while aDBS has been studied in just 45 patients across 7 published trials, the 18 ongoing studies are expected to include approximately 474 patients. Similarly, neurofeedback-based BCIs—currently supported by only four studies involving 22 participants—are projected to grow substantially, with 10 upcoming trials aiming to enroll an estimated 483 PD patients. These figures highlight the accelerating momentum in the field and the growing expectation that BCIs may soon contribute meaningfully to personalized, adaptive therapies for PD (Figure 2).

While these findings highlight the therapeutic promise of BCI technologies, they also underscore the need for broader investigation of non-invasive approaches and for larger, controlled studies to confirm their clinical efficacy and effectiveness.

### 7.1. Emerging Roles of Non-Invasive BCIs in PD

As non-invasive alternatives, eBCIs hold particular promise in addressing PD’s hallmark symptoms—bradykinesia, rigidity, and tremor—which are linked to abnormal oscillatory activity in motor circuits. These systems offer advantages in terms of safety, ease of use, and potential for home-based care, especially as PD management shifts toward personalized, adaptive, and continuous interventions [25].

For instance, MI-based BCIs harness preserved cortical excitability, activating the motor cortex and basal ganglia–thalamocortical circuits. Although concerns have been raised about whether these benefits can be achieved in patients with PD, studies have shown that MI paradigms can improve gait initiation, manual dexterity, and postural control, particularly when combined with EEG-guided feedback [53,63,64,91].

Neurofeedback approaches take it a step further by training users to modulate their neural activity consciously. Although historically used in epilepsy and ADHD, preliminary studies suggest benefits for PD in domains such as anxiety reduction, sleep improvement, and impulse control [1,3,92,93]. Hybrid BCIs integrating EMG or EOG sensors further enhance signal accuracy and responsiveness, which is critical for patients with dyskinesia, cognitive slowing, or fluctuating motor states [36].

### 7.2. The Future of eBCIs: Wearability, Accessibility, and Personalization

A key direction in BCI development is the integration of wearable neurotechnology. With the global wearables market reaching 534.6 million units in 2024 [94], the convergence of biosensing, mobile platforms, and machine learning is poised to transform PD care. Commercial-grade wireless EEG systems will have the capacity to detect cortical oscillations with sufficient resolution to inform closed-loop, patient-specific therapies [24,40,88].

However, electrode design remains a bottleneck in translating eBCIs to real-world clinical use. Optimal systems must strike a balance between comfort, signal fidelity, durability, and usability—particularly for patients with tremor, dyskinesia, or sensitive skin. Comparative studies are needed to evaluate wet, dry, and semi-dry electrode systems under realistic conditions, including variable sweating, motion artifacts, and long-term wearability [27].

Next-generation interfaces are exploring non-contact capacitive electrodes that can sense electrical fields through hair or clothing. Such solutions could enable seamless monitoring in daily life, especially for elderly or mobility-limited individuals. Integration into familiar wearable form factors—such as caps, headbands, or smart garments—could dramatically enhance user acceptance and adherence [27,90].

### 7.3. Ethical, Equity, and Security Considerations

As eBCI technologies advance, equity and usability must remain central concerns. Many individuals with PD face barriers related to cognitive impairment, comorbidities, or limited financial resources. The transition toward home-based neurorehabilitation will require simplified setups, intuitive interfaces, remote calibration, and embedded technical support—ensuring that BCIs are not only practical but also accessible and sustainable for long-term use [3,36,95].

Cybersecurity in BCIs is crucial due to risks such as unauthorized data access, device manipulation, and malware attacks. Because neural data is highly sensitive, strong encryption, secure authentication, and tamper-resistant designs are essential. Ensuring user privacy and ethical use also requires clear informed consent and regulatory oversight. As BCIs advance, protecting users must remain a top priority through continued interdisciplinary collaboration [96,97].

### 7.4. Ten Challenges in Parkinson’s Disease Treatment and Opportunities for BCIs

**Figure 3:**
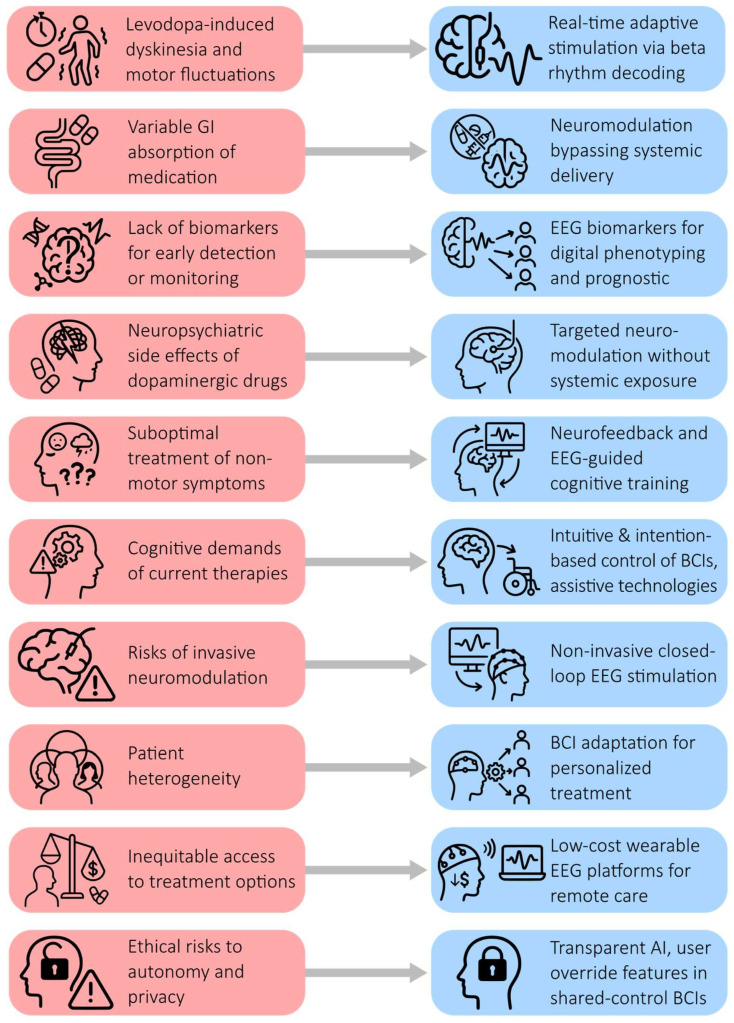
Challenges in Parkinson’s disease treatment and opportunities for BCIs.

Despite advances in pharmacological and surgical therapies, PD management continues to face significant challenges, particularly in later stages where conventional rehabilitation often loses efficacy due to fatigue, poor adherence, and declining mobility. BCIs, especially those that leverage electroencephalography, present a promising avenue for addressing these limitations by enabling adaptive, personalized, and non-invasive interventions. Enhanced by artificial intelligence, BCIs can decode complex motor intentions, facilitating high degrees of freedom in the control of prosthetic or assistive technology—a capability that may prove invaluable in mitigating levodopa-induced motor fluctuations [40]. Moreover, gamified interfaces and home-based BCI platforms offer innovative ways to sustain patient engagement and overcome barriers to long-term rehabilitation. Below, we outline ten critical challenges in current PD care and explore how BCIs could help overcome them (Figure 3).

A primary challenge is the high prevalence of motor fluctuations and levodopa-induced dyskinesia, which affect over 50% of patients after 5–10 years of treatment. Although newer formulations (e.g., IPX066) and infusion systems aim to reduce OFF time, they cannot dynamically adjust to rapid changes in neural activity [98]. BCIs, in contrast, can detect pathological oscillations in real time and support adaptive interventions that respond directly to the brain’s state [40].

Gastrointestinal issues represent a second challenge, particularly in advanced stages, where gastroparesis and dietary factors can impair the absorption of dopaminergic drugs. Even the most sophisticated delivery systems cannot bypass this bottleneck. BCIs offer a drug-free alternative by directly engaging motor circuits, potentially supporting neuromodulation without relying on systemic delivery.

The third, and arguably most pressing limitation, is the absence of disease-modifying therapies. Current treatments remain symptomatic. EEG biomarkers could aid in identifying early neurophysiological changes, facilitating patient stratification and redefining endpoints for neuroprotective trials. Closed-loop BCIs may enable early detection of decline and preemptive intervention before clinical symptoms worsen.

A fourth challenge is the neuropsychiatric side effects of dopaminergic drugs, including impulse control disorders, hallucinations, and mood disturbances. These effects are difficult to manage pharmacologically and often worsen quality of life. BCIs could provide targeted modulation of neural circuits implicated in mood and behavior, potentially reducing the risk of psychiatric complications, without systemic exposure.

The fifth challenge relates to the under-treatment of non-motor symptoms such as depression, fatigue, and executive dysfunction. These symptoms significantly affect quality of life but are often poorly addressed by conventional medications. Neurofeedback and EEG-based BCI for cognitive and motor training platforms, delivered through portable systems, show promise in targeting the neural correlates of these symptoms more directly.

As treatment complexity increases, the cognitive demands placed on patients represent a sixth challenge. Devices like deep brain stimulators and infusion pumps require intricate programming and patient engagement, which can be particularly difficult for those with cognitive impairments. BCIs offer intention-based control schemes that could simplify interaction with assistive technologies, thereby improving usability and adherence.

A seventh challenge is the surgical burden of invasive neuromodulation techniques. While deep brain stimulation and intestinal gel infusions are effective, their uptake is limited by procedural risks and access to healthcare. Non-invasive BCIs offer a scalable alternative that may expand access to individualized neuromodulation and rehabilitation.

PD’s marked phenotypic heterogeneity—the considerable variation in how the disease manifests and progresses across individuals—constitutes the eighth challenge. Patients differ not only in the type and severity of motor symptoms (e.g., tremor-dominant vs. akinetic-rigid forms) but also in the onset, presence, and degree of non-motor symptoms such as cognitive decline, mood disturbances, fatigue, and sleep dysfunction. Furthermore, the rate of disease progression and individual responses to pharmacological or surgical interventions vary widely, making it challenging to implement standardized therapeutic approaches that are effective for all patients [99,100,101]. By continuously monitoring and decoding neural activity, BCIs can adapt to individual neurophysiological profiles, allowing for more personalized and responsive interventions.

The ninth challenge is structural. Many promising neurotechnologies remain financially and logistically inaccessible in low- and middle-income settings. Current healthcare systems are not equipped to support AI- and neurotechnology-based platforms. Ensuring equitable access to BCI innovations will require rethinking reimbursement models and infrastructure to avoid exacerbating disparities.

The tenth and most complex challenge involves ethical concerns related to autonomy and user agency. As BCIs become capable of modulating behavior and cognition, questions arise regarding identity, consent, and control. Patients may misattribute actions to themselves or experience cognitive side effects. Addressing these risks requires a patient-centered design, continuous monitoring, and robust ethical oversight frameworks.

Despite these challenges, the future of BCI research in PD remains promising. Initiatives such as the U.S. BRAIN Initiative and the European Human Brain Project signal a growing commitment to ethical, collaborative neuroengineering. Realizing this potential will depend on interdisciplinary partnerships among clinicians, engineers, ethicists, and patient communities.

### 7.5. Barriers to Clinical Translation of BCIs in Parkinson’s Disease

Despite growing interest in BCIs for PD, several barriers continue to limit their clinical adoption. Technical challenges include low signal quality in non-invasive systems, susceptibility to artifacts, and the need for improved real-time processing and adaptive algorithms. Usability is also a concern, as many BCI paradigms require high levels of attention, training, and cognitive capacity, which can be difficult for individuals with advanced disease or fluctuating symptoms. Accessibility remains limited due to the high cost and infrastructure demands of some systems, such as aDBS. While wearable EEG devices offer a more scalable alternative, they currently lack the resolution and validation required for routine clinical use. Moreover, the clinical evidence base remains small, comprising only a few studies with heterogeneous designs and small sample sizes. Ethical concerns—such as the protection of neural data, device cybersecurity, and the need for explicit and informed consent—add further complexity. Finally, the lack of standardized regulatory pathways and limited integration with existing care models hinders broader implementation. Overcoming these challenges will be essential to realize the full potential of BCIs in Parkinson’s disease rehabilitation.

## 8. Conclusions

Current and emerging applications of BCIs in Parkinson’s disease span both invasive and non-invasive approaches for assistive and rehabilitation scenarios, with aDBS receiving the most attention. Although preliminary studies suggest potential benefits for motor and cognitive symptoms, the majority of available evidence is limited to small-scale pilot trials with heterogeneous designs. Key research directions include the development of real-time, user-centered systems, the integration of electrophysiological biomarkers for adaptive control, and the expansion of rehabilitation interventions. Broader clinical adoption will depend on resolving technical challenges, improving usability, validating clinical outcomes in larger cohorts, and addressing ethical considerations related to the use of neural data and patient autonomy. Continued interdisciplinary collaboration will be essential to translate BCIs into practical, personalized tools for PD rehabilitation.

This review is not exempt from the limitations inherent in a narrative review. Although a structured literature search was conducted, the review does not follow a systematic methodology or include a formal risk-of-bias assessment or quantitative synthesis of effect sizes. The selection of databases, while intended to balance clinical and technical coverage, may have omitted relevant studies not indexed within the chosen sources. Additionally, the early stage of research in this field and the methodological heterogeneity across studies limit the ability to draw definitive conclusions regarding clinical efficacy.

Despite these limitations, the evolving role of BCIs points toward a transformative future in PD care. EEG-based BCIs are not merely restoration tools but instruments of disruption and redefinition. By enabling continuous monitoring, adaptive treatment, and personalized rehabilitation, they challenge the episodic, clinic-bound model of care. Their rise offers an opportunity—not only to treat PD differently—but to reimagine what it means to live with it.

## Figures and Tables

**Table 1:** Comparison of BCI signal acquisition methods.

Type	Method	Temporal Resolution	Spatial Resolution	Long-Term Recording *	Portability *	Cost *	Safety *	Advantages	Disadvantages
Invasive	ECoG (incl. μECoG)	ms	mm	+++	++	++	−	Better SNR and resolution than EEG; less invasive than Utah probes.	Requires surgery; limited cortical coverage.
	SEEG	ms	mm	+	+	++	−	Records from deep brain structures; stable over time.	Surgical risks.
	Multi-single unit action potentials (e.g., Utah array)	µs–ms	µm	− −	+	+++	−	High-fidelity neuronal recordings; precise decoding.	Tissue damage; signal degradation over time.
	LFPs	ms	mm	+++	+++	++	+	Captures population-level dynamics; less sensitive to noise.	Lower resolution than a single unit; surgical risks.
Non-invasive	EEG	ms	cm	++	+++	−	+++	Cheap; portable; widely used.	Low spatial resolution; prone to artifacts.
	fNIRS	s	cm	++	+++	+	+++	Portable, safe, and valuable in infants and bedside settings.	Poor temporal resolution; limited to superficial cortex.
	fMRI	s	mm	− −	− −	+++	++	Excellent spatial resolution; whole-brain imaging.	Bulky; expensive; slow; not real-time.
	MEG	ms	mm–cm	+	−	+++	+++	Good spatial and temporal resolution.	An expensive, magnetically shielded room required.
	TCD	s	cm	+	++	+	+++	Inexpensive, portable, real-time blood flow measure.	Very low spatial resolution; indirect measure of brain activity.

* Evaluation parameters are qualitatively rated using a symbolic scale (“+++” = very high; “++” = high; “+” = moderate; “−” = low; “− −” = very low). These ratings reflect the authors’ subjective assessment based on literature and expert opinion and are intended for comparative illustration rather than quantitative accuracy. Abbreviations: ECoG, electrocorticography; μECoG, micro-ECoG; SEEG, stereoelectroencephalography; LFPs, local field potentials; EEG, electroencephalogram; fNIRS, functional near-infrared spectroscopy; fMRI, functional magnetic resonance imaging; MEG, magnetoencephalography; TCD, transcranial doppler ultrasonography; SNR, signal-to-noise ratio.

**Table 2:** Comparison of EEG-based BCI paradigms.

Paradigm	Type of Signal	Mental Workload *	Training Time	Possible applications in PD	Limitations
P300	Evoked	Moderate	Short (<1 h)	Spelling systems; attention monitoring.	Reduced performance in cases of visual or cognitive decline.
SSVEP	Evoked	Low	Short (<1 h)	Smart home control; assistive mobility.	Requires intact vision and gaze control.
Motor Imagery	Spontaneous	High	Long (>5 sessions)	Motor rehabilitation; neurofeedback.	High inter-subject variability; BCI illiteracy in some users.
Slow Cortical Potentials	Spontaneous	Moderate	Long (>5 sessions)	Binary communication or control in severe disability.	Low information transfer rate.
Hybrid (e.g., MI + SSVEP)	Mixed	High	Variable	High-dimensional control.	Complex configuration; risk of mental fatigue.

* Reflects a qualitative estimate of the attentional and mental effort typically required to use each paradigm, based on literature and expert opinion, to support relative comparison. It does not represent measured cognitive load. Abbreviations: P300, a positive event-related potential ~300 ms after a stimulus, linked to attention; SSVEP, steady-state visual evoked potentials, brain responses to repetitive visual stimuli at constant frequencies; MI, motor imagery.

## Data Availability

No new data were created or analyzed in this study. Data sharing does not apply to this article.

## Supplementary Materials

The following supporting information can be downloaded at: https://www.mdpi.com/article/10.3390/biomimetics10080488/s1, which includes details regarding the search strategy.

## Abbreviations

The following abbreviations are used in this manuscript:

ADHD	Attention-Deficit/Hyperactivity Disorder
AI	Artificial Intelligence
BCI	Brain–Computer Interface
CCA	Canonical Correlation Analysis
CNN	Convolutional Neural Network
CSP	Common Spatial Pattern
DBS	Deep Brain Stimulation
DL	Deep Learning
ECoG	Electrocorticography
EEG	Electroencephalography
EMG	Electromyography
EOG	Electrooculography
GAN	Generative Adversarial Network
ICA	Independent Component Analysis
LFP	Local Field Potential
MCI	Mild Cognitive Impairment
MEG	Magnetoencephalography
MEMD	Multivariate Empirical Mode Decomposition
MI	Motor Imagery
NclDBS	Neural Closed-Loop Deep Brain Stimulation
PAC	Phase–Amplitude Coupling
PD	Parkinson’s Disease
SCP	Slow Cortical Potential
SMA	Supplementary Motor Area
STN	Subthalamic Nucleus
TCD	Transcranial Doppler Ultrasonography
UPDRS	Unified Parkinson’s Disease Rating Scale
WT	Wavelet Transform
aDBS	Adaptive Deep Brain Stimulation
eBCI	EEG-based Brain–Computer Interface
fMRI	Functional Magnetic Resonance Imaging
fNIRS	Functional Near-Infrared Spectroscopy

## Appendix A

**Table A1:** ClinicalTrials.gov-registered studies on BCI use in Parkinson’s disease rehabilitation.

NCT Number	Study Title	Type of BCI	Purpose	Enrollment
NCT03422757	Safety and Efficacy of Adaptive DBS vs Conventional DBS in Patients With Parkinson’s Disease	aDBS	To assess safety and efficacy (in motor impairment and dyskinesia).	6 *
NCT02154724	Clinical Study for Adaptive Deep Brain Stimulation (aDBS) Controlled by Intracerebral Activity in Parkinson’s Disease	aDBS	To assess safety and efficacy (in motor impairment).	20 ^†^
NCT03724734	Trial of Adaptive Deep Brain Stimulation	aDBS	To assess safety and efficacy (in motor impairment during the day and night).	15 ^†^
NCT02384421	Adaptive Closed-Loop Neuromodulation and Neural Signatures of Parkinson’s Disease	aDBS	To assess efficacy (in motor symptoms, tremor, freezing of gait, bradykinesia).	22 *
NCT06891781	Investigating Adaptive Deep Brain Stimulation in Parkinson’s Disease Management	aDBS	To assess safety and efficacy (in motor and non-motor symptoms and quality of life).	72 ^†^
NCT04681534	Safety and Efficacy of Adaptive Deep Brain Stimulation	aDBS	To assess safety and efficacy (in motor impairment and dyskinesia).	15 ^†^
NCT05262348	An Open-Label Clinical Trial to Compare the Safety and Effectiveness of Adaptive versus Conventional Deep Brain Stimulation	aDBS	To assess safety and efficacy (in motor impairment).	0 *
NCT05402163	CANadian Adaptive DBS TriAl	aDBS	To assess efficacy (motor fluctuations, speech, gait impairment, and falls).	10 ^†^
NCT06909045	Adaptive vs. Continuous Subthalamic Nucleus Deep Brain Stimulation in Parkinson’s Disease	aDBS	To assess safety and efficacy (in motor and non-motor symptoms and quality of life).	130 ^†^
NCT06791902	Study on Preliminary Safety and Efficacy of Adaptive DBS Aligned to Locomotor States to Improve Locomotor Functions in Parkinson’s Patients	aDBS	To assess safety and efficacy (in locomotor function).	10 ^†^
NCT04547712	Adaptive DBS Algorithm for Personalized Therapy in Parkinson’s Disease	aDBS	To assess safety and efficacy (in motor impairment).	85 *
NCT04675398	Adaptive Deep Brain Stimulation to Improve Motor and Gait Functions in Parkinson’s Disease	aDBS	To assess safety and efficacy (in motor learning and gait function).	10 ^†^
NCT05070013	Adaptive Neurostimulation to Restore Sleep in Parkinson’s Disease (Aim 2)	aDBS	To assess efficacy (in sleep efficiency and quality).	20 ^†^
NCT02318927	A Responsive Closed-Loop Approach to Treat Freezing of Gait in Parkinson’s Disease	aDBS	To assess safety and efficacy (in motor function and freezing of gait).	8 *
NCT04620551	Adaptive Neurostimulation to Restore Sleep in Parkinson’s Disease	aDBS	To assess efficacy (in sleep efficiency and quality).	20 *
NCT06012461	Closed-Loop DBS in Parkinson’s Disease	aDBS	To assess long-term safety and efficacy (in motor function and sleep).	10 ^†^
NCT06819020	Adaptive Deep Brain Stimulation for Freezing of Gait in Parkinson’s Disease	aDBS	To assess efficacy (in motor function and freezing of gait).	20 ^†^
NCT03446833	LFP Beta aDBS Feasibility Study	aDBS	To assess safety and efficacy (in motor function, speech, and dyskinesia).	1 *
NCT06642519	Brain–Machine Interface for Freezing of Gait (Cortical Stimulation)	ECoG	To assess efficacy (in motor function and freezing of gait).	10 ^†^
NCT05696925	Effects of Motor Imagery and Action Observation on Upper Limb Motor Changes and Cognitive Changes in Parkinson’s Disease	EEG	To assess efficacy (in upper limb motor function and cognitive changes).	60 *
NCT06690931	Neurofeedback Rehabilitation With FES and VR for PD	EEG	To assess efficacy (in motor function and quality of life).	30 ^†^
NCT05986643	Brain Training to Improve Balance in Parkinson’s Disease	EEG	To assess efficacy (in balance and gait function).	100 ^†^
NCT04651478	Mental Representation Techniques for the Treatment of Parkinson’s Disease-Related Pain	EEG	To assess efficacy (in pain management and motor function).	32 ^†^
NCT05987865	Neurofeedback Training for PD	EEG/ LFPs	To assess efficacy (in motor symptoms and quality of life).	40 ^†^
NCT01867827	Real-Time fMRI Neurofeedback for Treatment of Parkinson’s Disease	fMRI	To assess efficacy (in motor function and brain activity modulation).	30 *
NCT06582355	FMRI-neurofeedback in Parkinson’s Disease	fMRI	To assess efficacy (in motor function and neuroplasticity).	60 ^†^
NCT03623386	Effect of Mental Imagery Training on Brain Plasticity and Motor Function in Individuals With Parkinson’s Disease	fMRI	To assess efficacy (in brain plasticity and motor function).	63 *
NCT05800470	The Effects of fNIRS-based Neurofeedback Training on Balance and Gait in Parkinson’s Disease	fNIRS	To assess efficacy (in balance and gait function).	48 ^†^
NCT03837548	A Study of Neurofeedback for the Treatment of Parkinson’s Disease	MEG	To assess efficacy (in motor function and brain activity modulation).	20 ^†^

* Actual enrollment. ^†^ Estimated enrollment. Abbreviations: PD, Parkinson’s disease; EEG, electroencephalography; ECoG, electrocorticography; LFPs, local field potentials; fMRI, functional magnetic resonance imaging; fNIRS, functional near-infrared spectroscopy; MEG, magnetoencephalography; BCI, brain–computer interface; DBS, deep brain stimulation; aDBS, adaptive DBS.
