# Supplementary material for: Brain–Computer Interfaces in Parkinson’s Disease Rehabilitation
Source: Biomimetics (Basel). 2025 Jul 23;10(8):488. doi: 10.3390/biomimetics10080488 (PMC12383679; doi:10.3390/biomimetics10080488)

Brain–Computer Interfaces for Parkinson's Disease Rehabilitation

Supplementary Material

Search strategy

We included articles published in peer-reviewed journals up to May 2025, regardless of language.

Inclusion criteria: Original clinical research articles focused on the use of a BCI or neural implant in Parkinson's disease patients for rehabilitation of motor or non-motor symptoms.

Exclusion criteria: Reviews and studies with poorly defined methodologies or insufficient data to support conclusions.

Search query in PubMed database:

("brain computer interface"[Title/Abstract] OR "BCI"[Title/Abstract] OR "Brain-Machine Interface"[Title/Abstract] OR "Neural Interface"[Title/Abstract] OR "Neural Prosthetics"[Title/Abstract] OR "Brain-Computer Communication"[Title/Abstract] OR "Cortical Interfaces"[Title/Abstract] OR "Neurointerfaces"[Title/Abstract] OR "Brain-Computer Interfaces"[MeSH]) AND ("parkinson's disease") AND ("Rehabilitation"[MeSH] OR "rehabilitation"[Title/Abstract] OR "therapy"[Title/Abstract] OR "Treatment"[Title/Abstract] OR "Rehabilitation"[Mesh] OR "Physical Therapy Modalities"[Mesh] OR "Exercise Therapy"[Mesh] OR "Neurological Rehabilitation"[Mesh] OR "Occupational Therapy"[Mesh] OR "Treatment Outcome"[Mesh] OR "Recovery of Function"[Mesh])

Search query in Web of Science database:

TS=("brain computer interface" OR BCI OR "brain-machine interface" OR "neural interface" OR "neural-control interface" OR "mind-machine interface" OR "direct neural interface" OR "neural prosthetics" OR "brain-computer communication" OR "cortical interfaces" OR neurointerfaces OR "brain-computer interfaces") AND TS=("parkinson's disease") AND TS=(rehabilitation OR therapy OR treatment OR "physical therapy" OR "exercise therapy" OR "neurological rehabilitation" OR "occupational therapy" OR "treatment outcome" OR "recovery of function" OR "functional recovery" OR "motor rehabilitation" OR "neurorehabilitation" OR "motor learning" OR "task-oriented training" OR "functional training")

Search query in Scopus database:

(TITLE-ABS-KEY("brain computer interface" OR BCI OR "brain-machine interface" OR "neural interface" OR "neural-control interface" OR "mind-machine interface" OR "direct neural interface" OR "neural prosthetics" OR "brain-computer communication" OR "cortical interfaces" OR neurointerfaces OR "brain-computer interfaces")) AND (TITLE-ABS-KEY("parkinson's disease")) AND (TITLE-ABS-KEY(rehabilitation OR therapy OR treatment OR "physical therapy" OR "exercise therapy" OR "neurological rehabilitation" OR "occupational therapy" OR "treatment outcome" OR "recovery of function" OR "functional

recovery" OR "motor rehabilitation" OR neurorehabilitation OR "motor learning" OR "task-oriented training" OR "functional training"))

Search query in Google Scholar:

("brain computer interface" OR "brain-machine interface" OR "neural interface" OR "neural prosthetics" OR "cortical interface") AND intitle:parkinson AND ("rehabilitation" OR "therapy" OR "treatment" OR "recovery of function" OR "functional recovery" OR "neurorehabilitation" OR "motor learning")

Search query in IEEE Xplore:

(("All Metadata":Brain computer interface) OR ("All Metadata":brain machine interface) OR ("All Metadata":neural interface) OR ("All Metadata":neural prosthetics) OR ("All Metadata":cortical interface)) AND ("All Metadata":parkinson) AND (("All Metadata":rehabilitation) OR ("All Metadata":therapy) OR ("All Metadata":treatment) OR ("All Metadata":recovery of function) OR ("All Metadata":functional recovery) OR ("All Metadata":neurorehabilitation) OR ("All Metadata":motor learning))

Flow diagram:

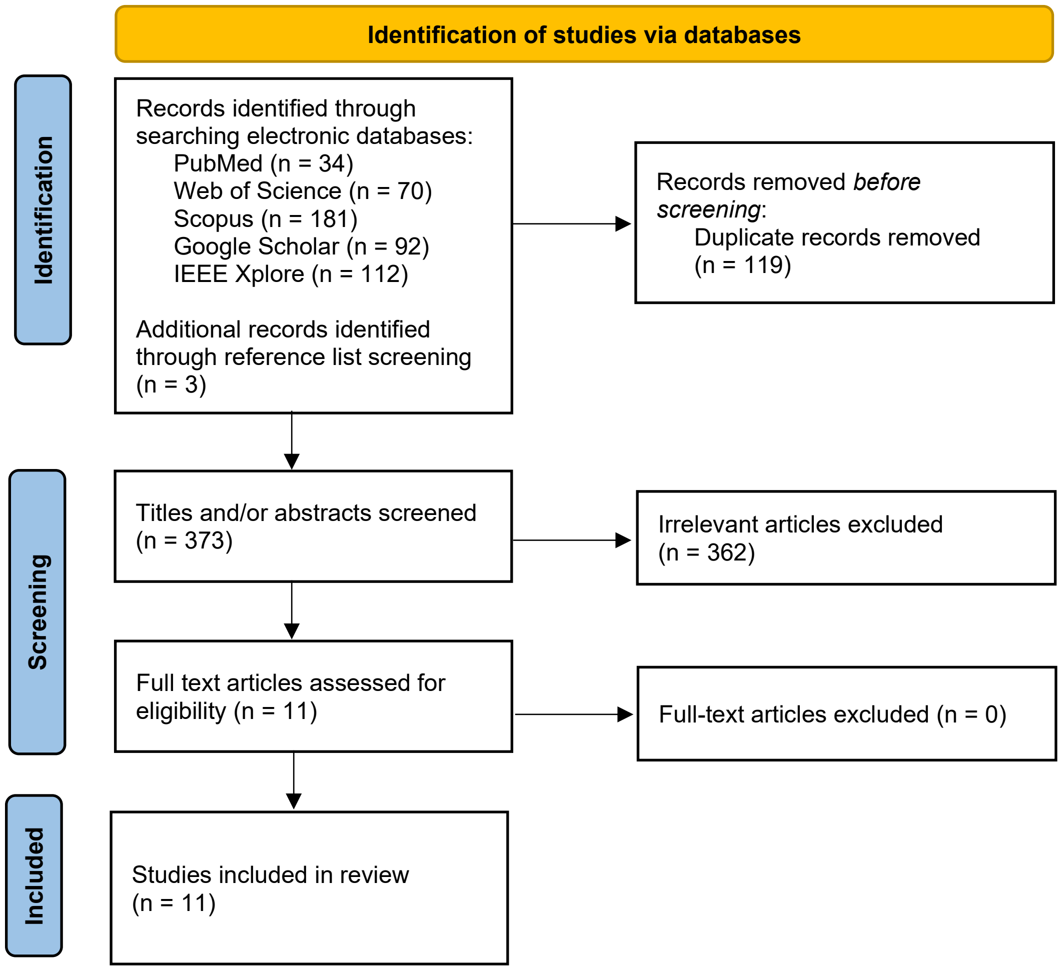

Supplement: Supplementary file 1 [file biomimetics-10-00488-s001.zip › biomimetics-3707738-supplementary.pdf]
